# 1,1′-Bis(*tert*-butyl­dimethyl­sil­yl)ferrocene

**DOI:** 10.1107/S1600536812049069

**Published:** 2012-12-12

**Authors:** Abdolreza Abri, Behzad Soltani, Christopher J. Ziegler, James T. Engle, Reza Kia

**Affiliations:** a Department of Chemistry, Azarbaijan Shahid Madani University, Tabriz, Iran; b Department of Chemistry, University of Akron, Akron, OH, USA; c Department of Chemistry, Science and Rsearch Branch, Islamic Azad University, Tehran, Iran

## Abstract

The asymmetric unit of the title compound, [Fe(C_11_H_19_Si)_2_], consists of one half of a ferrocene derivative. The Fe^II^ atom lies on a twofold rotation axis, giving an eclipsed conformation for the cyclo­penta­dienyl ligands. No significant inter­molecular inter­actions are observed in the crystal structure.

## Related literature

For background to ferrocene derivatives and their applications, see: Hudson *et al.* (2001 ▶); Liu *et al.* (2000 ▶). For a related structure, see: Ren *et al.* (2012 ▶).
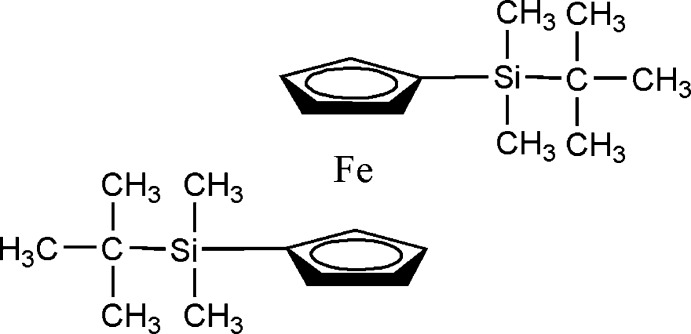


## Experimental

### 

#### Crystal data

[Fe(C_11_H_19_Si)_2_]
*M* *_r_* = 414.55
Orthorhombic, 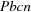

*a* = 7.1282 (6) Å
*b* = 12.1466 (10) Å
*c* = 26.363 (2) Å
*V* = 2282.6 (3) Å^3^
*Z* = 4
Mo *K*α radiation
μ = 0.77 mm^−1^
*T* = 100 K
0.20 × 0.20 × 0.10 mm

#### Data collection

Bruker APEXII CCD diffractometer
Absorption correction: multi-scan (*SADABS*; Bruker, 2005 ▶) *T*_min_ = 0.861, *T*_max_ = 0.927
17220 measured reflections
2529 independent reflections
2323 reflections with *I* > 2σ(*I*)
*R*_int_ = 0.037

#### Refinement

*R*[*F* ^2^ > 2σ(*F* ^2^)] = 0.026
*wR*(*F* ^2^) = 0.067
*S* = 1.05
2529 reflections
114 parameters
H-atom parameters constrained
Δρ_max_ = 0.36 e Å^−3^
Δρ_min_ = −0.24 e Å^−3^

### 

Data collection: *APEX2* (Bruker, 2005 ▶); cell refinement: *SAINT* (Bruker, 2005 ▶); data reduction: *SAINT*; program(s) used to solve structure: *SHELXS97* (Sheldrick, 2008 ▶); program(s) used to refine structure: *SHELXL97* (Sheldrick, 2008 ▶); molecular graphics: *SHELXTL* (Sheldrick, 2008 ▶); software used to prepare material for publication: *SHELXTL* and *PLATON* (Spek, 2009 ▶).

## Supplementary Material

Crystal structure: contains datablock(s) global, I. DOI: 10.1107/S1600536812049069/is5223sup1.cif

Structure factors: contains datablock(s) I. DOI: 10.1107/S1600536812049069/is5223Isup2.hkl

Additional supplementary materials:  crystallographic information; 3D view; checkCIF report

## Figures and Tables

**Fig. 1.**
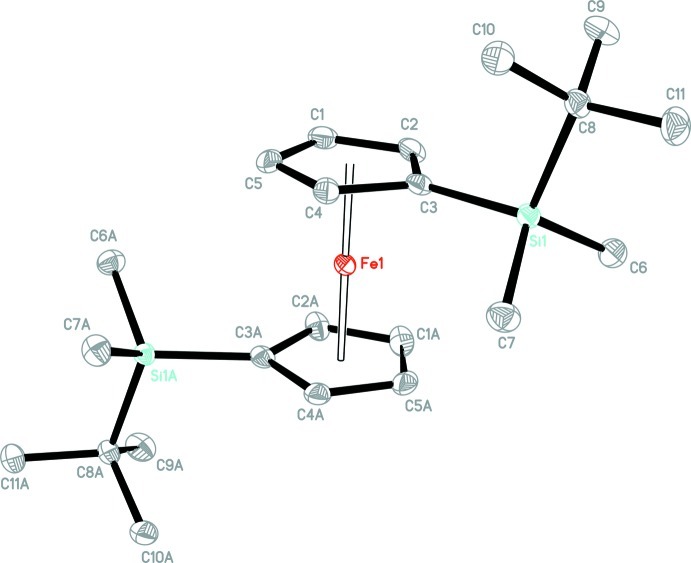
The ORTEP plot of the title compound, showing 40% probability displacement ellipsoids and the atomic numbering [symmetry code for suffix A: -x + 2, y, -z + 1/2]. The H atoms were omitted for clarity.

## supplementary crystallographic information

### Comment

Ferrocene has attracted the interest of many scientists and research groups worldwide because of its applications in materials science (Hudson *et al.*, 2001; Liu *et al.*, 2000). Ferrocene as a starting material in synthetic organometallic systems and its derivatives in industrial applications have become a great area of interest for many researchers and industrial chemists.

The asymmetric unit of the title compound comprises a half of a ferrocene drivative (Fig. 1). The Fe^II^ atom lies on a twofold rotation axis, giving an eclipsed conformation for the cyclopentadienyl ligands. The bond lengths and angles are within the normal ranges and are comparable to the related structure (Ren *et al.*, 2012).

### Experimental

To a stirred solution of ferrocene (5.00 g, 26.88 mmol) in 100 ml of *n*-hexane, a solution containing 9.2 ml (60 mmol) tetramethylethylenediamine (tmeda) and 35 ml (56 mmol) of a 1.6 *M* of *n*-BuLi in 30 ml of *n*-hexane was added dropwise over 5 min, and mixture was stirred overnight. The orange precipitate of FcLi_2_ was collected. A THF solution (20 ml) containing FcLi_2_ (0.67 g, 3.5 mmol) was added dropwise to a THF solution (10 ml) of 1.67 ml *t*-butyldimethylchlorosilane (13.9 mmol) at -30 °C, and were kept for 30 min at -30 °C and then stirred over night at room temperature. The solvent THF was evaporated together with the excess of chlorosilane under vacuum, then, the orange residue dissolved in n-hexane (30 ml) and the solution filtered over Na_2_SO_4_. The solvent *n*-hexane was removed under vacuum and the product heated up for 1 h in order to sublime the impurities of ferrocene off. The remaining dark-orange oil or solid (0.69 g, 55% yield) was obtained. Single Crystals suitable for X-ray analysis were obtained by slow evaporation from a *n*-hexane solution at room temperature.

### Refinement

All hydrogen atoms were positioned geometrically with C—H = 0.95 or 0.98 Å and included in a riding model approximation with *U*_iso_ (H) = 1.2*U*_eq_(C).

### Crystal data

**Table:**

[Fe(C_11_H_19_Si)_2_]	*F*(000) = 896
*M**_r_* = 414.55	*D*_x_ = 1.206 Mg m^−^^3^
Orthorhombic, *P**b**c**n*	Mo *K*α radiation, λ = 0.71073 Å
Hall symbol: -P 2n 2ab	Cell parameters from 9976 reflections
*a* = 7.1282 (6) Å	θ = 2.3–27.2°
*b* = 12.1466 (10) Å	µ = 0.77 mm^−^^1^
*c* = 26.363 (2) Å	*T* = 100 K
*V* = 2282.6 (3) Å^3^	Block, orange
*Z* = 4	0.20 × 0.20 × 0.10 mm

### Data collection

**Table:**

Bruker APEXII CCD diffractometer	2529 independent reflections
Radiation source: fine-focus sealed tube	2323 reflections with *I* > 2σ(*I*)
Graphite monochromator	*R*_int_ = 0.037
φ and ω scans	θ_max_ = 27.2°, θ_min_ = 3.1°
Absorption correction: multi-scan (*SADABS*; Bruker, 2005)	*h* = −9→9
*T*_min_ = 0.861, *T*_max_ = 0.927	*k* = −15→15
17220 measured reflections	*l* = −33→33

### Refinement

**Table:**

Refinement on *F*^2^	Primary atom site location: structure-invariant direct methods
Least-squares matrix: full	Secondary atom site location: difference Fourier map
*R*[*F*^2^ > 2σ(*F*^2^)] = 0.026	Hydrogen site location: inferred from neighbouring sites
*wR*(*F*^2^) = 0.067	H-atom parameters constrained
*S* = 1.05	*w* = 1/[σ^2^(*F*_o_^2^) + (0.0312*P*)^2^ + 1.1338*P*] where *P* = (*F*_o_^2^ + 2*F*_c_^2^)/3
2529 reflections	(Δ/σ)_max_ = 0.001
114 parameters	Δρ_max_ = 0.36 e Å^−^^3^
0 restraints	Δρ_min_ = −0.24 e Å^−^^3^

### Special details

**Table:**

Geometry. All e.s.d.'s (except the e.s.d. in the dihedral angle between two l.s. planes) are estimated using the full covariance matrix. The cell e.s.d.'s are taken into account individually in the estimation of e.s.d.'s in distances, angles and torsion angles; correlations between e.s.d.'s in cell parameters are only used when they are defined by crystal symmetry. An approximate (isotropic) treatment of cell e.s.d.'s is used for estimating e.s.d.'s involving l.s. planes.
Refinement. Refinement of *F*^2^ against ALL reflections. The weighted *R*-factor *wR* and goodness of fit *S* are based on *F*^2^, conventional *R*-factors *R* are based on *F*, with *F* set to zero for negative *F*^2^. The threshold expression of *F*^2^ > σ(*F*^2^) is used only for calculating *R*-factors(gt) *etc*. and is not relevant to the choice of reflections for refinement. *R*-factors based on *F*^2^ are statistically about twice as large as those based on *F*, and *R*- factors based on ALL data will be even larger.

### Fractional atomic coordinates and isotropic or equivalent isotropic displacement parameters (Å^2^)

**Table:**

	*x*	*y*	*z*	*U*_iso_*/*U*_eq_	
Fe1	1.0000	0.03916 (2)	0.2500	0.01182 (9)	
Si1	0.96501 (5)	0.11765 (3)	0.122364 (13)	0.01274 (10)	
C1	0.7486 (2)	−0.04225 (12)	0.24866 (5)	0.0212 (3)	
H1	0.7161	−0.1051	0.2682	0.025*	
C2	0.8338 (2)	−0.04320 (11)	0.19970 (5)	0.0181 (3)	
H2	0.8674	−0.1072	0.1811	0.022*	
C3	0.86070 (18)	0.06855 (10)	0.18305 (5)	0.0143 (3)	
C4	0.78856 (18)	0.13706 (11)	0.22318 (5)	0.0167 (3)	
H4	0.7866	0.2153	0.2230	0.020*	
C5	0.72076 (18)	0.06891 (12)	0.26299 (5)	0.0197 (3)	
H5	0.6663	0.0936	0.2938	0.024*	
C6	1.14332 (19)	0.01654 (11)	0.09925 (5)	0.0198 (3)	
H6A	1.2446	0.0103	0.1242	0.030*	
H6B	1.1949	0.0419	0.0668	0.030*	
H6C	1.0839	−0.0555	0.0946	0.030*	
C7	1.0752 (2)	0.25562 (11)	0.13317 (5)	0.0203 (3)	
H7A	1.1764	0.2486	0.1581	0.030*	
H7B	0.9803	0.3071	0.1459	0.030*	
H7C	1.1266	0.2833	0.1012	0.030*	
C8	0.76825 (18)	0.13000 (10)	0.07396 (5)	0.0156 (3)	
C9	0.6877 (2)	0.01524 (12)	0.06271 (5)	0.0231 (3)	
H9A	0.5864	0.0216	0.0378	0.035*	
H9B	0.6384	−0.0170	0.0941	0.035*	
H9D	0.7870	−0.0321	0.0491	0.035*	
C10	0.61107 (19)	0.20356 (12)	0.09496 (5)	0.0212 (3)	
H10A	0.5103	0.2095	0.0698	0.032*	
H10D	0.6613	0.2770	0.1022	0.032*	
H10B	0.5613	0.1711	0.1262	0.032*	
C11	0.8419 (2)	0.18068 (12)	0.02429 (5)	0.0233 (3)	
H11D	0.7389	0.1864	−0.0002	0.035*	
H11A	0.9409	0.1337	0.0103	0.035*	
H11B	0.8927	0.2542	0.0311	0.035*	

### Atomic displacement parameters (Å^2^)

**Table:**

	*U*^11^	*U*^22^	*U*^33^	*U*^12^	*U*^13^	*U*^23^
Fe1	0.01172 (14)	0.01365 (14)	0.01008 (14)	0.000	−0.00130 (9)	0.000
Si1	0.01328 (17)	0.01321 (18)	0.01173 (17)	−0.00049 (13)	−0.00074 (13)	0.00000 (12)
C1	0.0187 (7)	0.0279 (8)	0.0170 (7)	−0.0100 (6)	−0.0031 (5)	0.0032 (5)
C2	0.0212 (7)	0.0189 (7)	0.0142 (6)	−0.0060 (5)	−0.0039 (5)	−0.0003 (5)
C3	0.0130 (6)	0.0168 (6)	0.0130 (6)	−0.0010 (5)	−0.0042 (5)	−0.0004 (5)
C4	0.0135 (6)	0.0221 (7)	0.0146 (6)	0.0036 (5)	−0.0030 (5)	−0.0009 (5)
C5	0.0107 (6)	0.0339 (8)	0.0145 (6)	0.0004 (6)	−0.0010 (5)	−0.0009 (5)
C6	0.0175 (6)	0.0218 (7)	0.0202 (7)	0.0021 (6)	0.0002 (5)	−0.0022 (5)
C7	0.0217 (7)	0.0179 (7)	0.0212 (7)	−0.0034 (6)	−0.0016 (6)	0.0004 (5)
C8	0.0171 (6)	0.0177 (6)	0.0121 (6)	0.0005 (5)	−0.0012 (5)	0.0011 (5)
C9	0.0236 (7)	0.0245 (7)	0.0213 (7)	−0.0034 (6)	−0.0079 (6)	−0.0021 (5)
C10	0.0185 (7)	0.0271 (7)	0.0179 (6)	0.0051 (6)	−0.0019 (5)	0.0022 (5)
C11	0.0244 (7)	0.0309 (8)	0.0146 (6)	0.0029 (6)	0.0009 (6)	0.0043 (5)

### Geometric parameters (Å, º)

**Table:**

Fe1—C2^i^	2.0401 (13)	C4—H4	0.9500
Fe1—C2	2.0402 (13)	C5—H5	0.9500
Fe1—C4	2.0460 (13)	C6—H6A	0.9800
Fe1—C4^i^	2.0460 (13)	C6—H6B	0.9800
Fe1—C1^i^	2.0473 (15)	C6—H6C	0.9800
Fe1—C1	2.0473 (15)	C7—H7A	0.9800
Fe1—C5^i^	2.0518 (13)	C7—H7B	0.9800
Fe1—C5	2.0518 (13)	C7—H7C	0.9800
Fe1—C3	2.0564 (12)	C8—C9	1.5363 (19)
Fe1—C3^i^	2.0565 (12)	C8—C10	1.5364 (18)
Si1—C3	1.8622 (13)	C8—C11	1.5391 (17)
Si1—C6	1.8696 (14)	C9—H9A	0.9800
Si1—C7	1.8726 (14)	C9—H9B	0.9800
Si1—C8	1.9022 (13)	C9—H9D	0.9800
C1—C5	1.416 (2)	C10—H10A	0.9800
C1—C2	1.4267 (19)	C10—H10D	0.9800
C1—H1	0.9500	C10—H10B	0.9800
C2—C3	1.4394 (17)	C11—H11D	0.9800
C2—H2	0.9500	C11—H11A	0.9800
C3—C4	1.4409 (17)	C11—H11B	0.9800
C4—C5	1.4214 (19)		
			
C2^i^—Fe1—C2	121.27 (8)	C3—C2—Fe1	70.04 (7)
C2^i^—Fe1—C4	159.61 (5)	C1—C2—H2	125.5
C2—Fe1—C4	68.45 (5)	C3—C2—H2	125.5
C2^i^—Fe1—C4^i^	68.45 (5)	Fe1—C2—H2	126.2
C2—Fe1—C4^i^	159.61 (5)	C2—C3—C4	105.85 (11)
C4—Fe1—C4^i^	108.92 (8)	C2—C3—Si1	128.11 (10)
C2^i^—Fe1—C1^i^	40.86 (5)	C4—C3—Si1	126.04 (10)
C2—Fe1—C1^i^	106.42 (6)	C2—C3—Fe1	68.82 (7)
C4—Fe1—C1^i^	158.69 (5)	C4—C3—Fe1	69.05 (7)
C4^i^—Fe1—C1^i^	68.27 (6)	Si1—C3—Fe1	126.88 (7)
C2^i^—Fe1—C1	106.42 (6)	C5—C4—C3	109.09 (12)
C2—Fe1—C1	40.86 (5)	C5—C4—Fe1	69.92 (7)
C4—Fe1—C1	68.27 (6)	C3—C4—Fe1	69.83 (7)
C4^i^—Fe1—C1	158.69 (5)	C5—C4—H4	125.5
C1^i^—Fe1—C1	122.24 (9)	C3—C4—H4	125.5
C2^i^—Fe1—C5^i^	68.38 (6)	Fe1—C4—H4	126.4
C2—Fe1—C5^i^	122.76 (6)	C1—C5—C4	108.11 (12)
C4—Fe1—C5^i^	123.69 (6)	C1—C5—Fe1	69.62 (8)
C4^i^—Fe1—C5^i^	40.59 (5)	C4—C5—Fe1	69.48 (7)
C1^i^—Fe1—C5^i^	40.42 (6)	C1—C5—H5	125.9
C1—Fe1—C5^i^	158.67 (6)	C4—C5—H5	125.9
C2^i^—Fe1—C5	122.76 (6)	Fe1—C5—H5	126.5
C2—Fe1—C5	68.38 (6)	Si1—C6—H6A	109.5
C4—Fe1—C5	40.59 (5)	Si1—C6—H6B	109.5
C4^i^—Fe1—C5	123.68 (6)	H6A—C6—H6B	109.5
C1^i^—Fe1—C5	158.67 (6)	Si1—C6—H6C	109.5
C1—Fe1—C5	40.42 (6)	H6A—C6—H6C	109.5
C5^i^—Fe1—C5	159.71 (9)	H6B—C6—H6C	109.5
C2^i^—Fe1—C3	157.42 (5)	Si1—C7—H7A	109.5
C2—Fe1—C3	41.14 (5)	Si1—C7—H7B	109.5
C4—Fe1—C3	41.13 (5)	H7A—C7—H7B	109.5
C4^i^—Fe1—C3	123.47 (5)	Si1—C7—H7C	109.5
C1^i^—Fe1—C3	121.43 (5)	H7A—C7—H7C	109.5
C1—Fe1—C3	69.29 (5)	H7B—C7—H7C	109.5
C5^i^—Fe1—C3	107.13 (5)	C9—C8—C10	108.95 (11)
C5—Fe1—C3	69.16 (5)	C9—C8—C11	109.03 (11)
C2^i^—Fe1—C3^i^	41.14 (5)	C10—C8—C11	108.82 (11)
C2—Fe1—C3^i^	157.42 (5)	C9—C8—Si1	109.48 (9)
C4—Fe1—C3^i^	123.46 (5)	C10—C8—Si1	109.98 (9)
C4^i^—Fe1—C3^i^	41.13 (5)	C11—C8—Si1	110.54 (9)
C1^i^—Fe1—C3^i^	69.29 (5)	C8—C9—H9A	109.5
C1—Fe1—C3^i^	121.42 (5)	C8—C9—H9B	109.5
C5^i^—Fe1—C3^i^	69.16 (5)	H9A—C9—H9B	109.5
C5—Fe1—C3^i^	107.13 (5)	C8—C9—H9D	109.5
C3—Fe1—C3^i^	160.00 (7)	H9A—C9—H9D	109.5
C3—Si1—C6	109.94 (6)	H9B—C9—H9D	109.5
C3—Si1—C7	108.87 (6)	C8—C10—H10A	109.5
C6—Si1—C7	110.63 (6)	C8—C10—H10D	109.5
C3—Si1—C8	107.89 (6)	H10A—C10—H10D	109.5
C6—Si1—C8	109.53 (6)	C8—C10—H10B	109.5
C7—Si1—C8	109.93 (6)	H10A—C10—H10B	109.5
C5—C1—C2	107.98 (12)	H10D—C10—H10B	109.5
C5—C1—Fe1	69.96 (8)	C8—C11—H11D	109.5
C2—C1—Fe1	69.30 (8)	C8—C11—H11A	109.5
C5—C1—H1	126.0	H11D—C11—H11A	109.5
C2—C1—H1	126.0	C8—C11—H11B	109.5
Fe1—C1—H1	126.3	H11D—C11—H11B	109.5
C1—C2—C3	108.96 (12)	H11A—C11—H11B	109.5
C1—C2—Fe1	69.84 (8)		
			
C2^i^—Fe1—C1—C5	121.60 (8)	C5—Fe1—C3—C4	36.93 (8)
C2—Fe1—C1—C5	−119.28 (11)	C3^i^—Fe1—C3—C4	−45.93 (7)
C4—Fe1—C1—C5	−37.55 (8)	C2^i^—Fe1—C3—Si1	−76.71 (17)
C4^i^—Fe1—C1—C5	48.96 (18)	C2—Fe1—C3—Si1	−122.46 (12)
C1^i^—Fe1—C1—C5	163.33 (9)	C4—Fe1—C3—Si1	120.02 (12)
C5^i^—Fe1—C1—C5	−165.93 (10)	C4^i^—Fe1—C3—Si1	39.57 (11)
C3—Fe1—C1—C5	−81.80 (8)	C1^i^—Fe1—C3—Si1	−43.76 (11)
C3^i^—Fe1—C1—C5	79.33 (9)	C1—Fe1—C3—Si1	−159.69 (10)
C2^i^—Fe1—C1—C2	−119.12 (9)	C5^i^—Fe1—C3—Si1	−1.95 (10)
C4—Fe1—C1—C2	81.73 (8)	C5—Fe1—C3—Si1	156.94 (10)
C4^i^—Fe1—C1—C2	168.24 (14)	C3^i^—Fe1—C3—Si1	74.08 (7)
C1^i^—Fe1—C1—C2	−77.39 (8)	C2—C3—C4—C5	0.22 (14)
C5^i^—Fe1—C1—C2	−46.65 (18)	Si1—C3—C4—C5	179.86 (9)
C5—Fe1—C1—C2	119.28 (11)	Fe1—C3—C4—C5	−59.06 (9)
C3—Fe1—C1—C2	37.48 (8)	C2—C3—C4—Fe1	59.28 (9)
C3^i^—Fe1—C1—C2	−161.39 (8)	Si1—C3—C4—Fe1	−121.08 (10)
C5—C1—C2—C3	0.20 (16)	C2^i^—Fe1—C4—C5	−41.15 (19)
Fe1—C1—C2—C3	−59.29 (10)	C2—Fe1—C4—C5	81.50 (9)
C5—C1—C2—Fe1	59.49 (10)	C4^i^—Fe1—C4—C5	−120.06 (9)
C2^i^—Fe1—C2—C1	78.66 (8)	C1^i^—Fe1—C4—C5	161.35 (15)
C4—Fe1—C2—C1	−81.27 (9)	C1—Fe1—C4—C5	37.39 (8)
C4^i^—Fe1—C2—C1	−167.72 (15)	C5^i^—Fe1—C4—C5	−162.65 (8)
C1^i^—Fe1—C2—C1	120.62 (11)	C3—Fe1—C4—C5	120.35 (11)
C5^i^—Fe1—C2—C1	161.66 (9)	C3^i^—Fe1—C4—C5	−76.78 (10)
C5—Fe1—C2—C1	−37.47 (8)	C2^i^—Fe1—C4—C3	−161.50 (15)
C3—Fe1—C2—C1	−120.11 (12)	C2—Fe1—C4—C3	−38.85 (8)
C3^i^—Fe1—C2—C1	45.19 (18)	C4^i^—Fe1—C4—C3	119.58 (8)
C2^i^—Fe1—C2—C3	−161.23 (9)	C1^i^—Fe1—C4—C3	41.00 (19)
C4—Fe1—C2—C3	38.84 (8)	C1—Fe1—C4—C3	−82.96 (8)
C4^i^—Fe1—C2—C3	−47.61 (19)	C5^i^—Fe1—C4—C3	76.99 (9)
C1^i^—Fe1—C2—C3	−119.27 (8)	C5—Fe1—C4—C3	−120.35 (11)
C1—Fe1—C2—C3	120.11 (12)	C3^i^—Fe1—C4—C3	162.87 (7)
C5^i^—Fe1—C2—C3	−78.22 (10)	C2—C1—C5—C4	−0.06 (16)
C5—Fe1—C2—C3	82.65 (8)	Fe1—C1—C5—C4	59.01 (9)
C3^i^—Fe1—C2—C3	165.30 (9)	C2—C1—C5—Fe1	−59.08 (10)
C1—C2—C3—C4	−0.26 (15)	C3—C4—C5—C1	−0.10 (15)
Fe1—C2—C3—C4	−59.42 (8)	Fe1—C4—C5—C1	−59.10 (10)
C1—C2—C3—Si1	−179.89 (10)	C3—C4—C5—Fe1	59.00 (9)
Fe1—C2—C3—Si1	120.94 (11)	C2^i^—Fe1—C5—C1	−76.28 (9)
C1—C2—C3—Fe1	59.17 (10)	C2—Fe1—C5—C1	37.86 (8)
C6—Si1—C3—C2	−30.07 (14)	C4—Fe1—C5—C1	119.54 (11)
C7—Si1—C3—C2	−151.41 (12)	C4^i^—Fe1—C5—C1	−160.77 (7)
C8—Si1—C3—C2	89.33 (13)	C1^i^—Fe1—C5—C1	−41.8 (2)
C6—Si1—C3—C4	150.36 (11)	C5^i^—Fe1—C5—C1	165.22 (8)
C7—Si1—C3—C4	29.03 (13)	C3—Fe1—C5—C1	82.14 (8)
C8—Si1—C3—C4	−90.24 (12)	C3^i^—Fe1—C5—C1	−118.66 (8)
C6—Si1—C3—Fe1	60.76 (9)	C2^i^—Fe1—C5—C4	164.18 (8)
C7—Si1—C3—Fe1	−60.57 (10)	C2—Fe1—C5—C4	−81.67 (8)
C8—Si1—C3—Fe1	−179.84 (7)	C4^i^—Fe1—C5—C4	79.69 (11)
C2^i^—Fe1—C3—C2	45.8 (2)	C1^i^—Fe1—C5—C4	−161.38 (13)
C4—Fe1—C3—C2	−117.52 (11)	C1—Fe1—C5—C4	−119.54 (11)
C4^i^—Fe1—C3—C2	162.04 (8)	C5^i^—Fe1—C5—C4	45.68 (7)
C1^i^—Fe1—C3—C2	78.70 (10)	C3—Fe1—C5—C4	−37.39 (8)
C1—Fe1—C3—C2	−37.23 (8)	C3^i^—Fe1—C5—C4	121.81 (8)
C5^i^—Fe1—C3—C2	120.52 (9)	C3—Si1—C8—C9	−65.20 (10)
C5—Fe1—C3—C2	−80.59 (9)	C6—Si1—C8—C9	54.46 (11)
C3^i^—Fe1—C3—C2	−163.45 (8)	C7—Si1—C8—C9	176.22 (9)
C2^i^—Fe1—C3—C4	163.27 (13)	C3—Si1—C8—C10	54.48 (10)
C2—Fe1—C3—C4	117.52 (11)	C6—Si1—C8—C10	174.14 (9)
C4^i^—Fe1—C3—C4	−80.44 (11)	C7—Si1—C8—C10	−64.10 (11)
C1^i^—Fe1—C3—C4	−163.78 (8)	C3—Si1—C8—C11	174.68 (9)
C1—Fe1—C3—C4	80.29 (8)	C6—Si1—C8—C11	−65.67 (11)
C5^i^—Fe1—C3—C4	−121.96 (8)	C7—Si1—C8—C11	56.09 (11)

Symmetry code: (i) −*x*+2, *y*, −*z*+1/2.
